# Mitochondrial chaotic dynamics: Redox-energetic behavior at the edge of stability

**DOI:** 10.1038/s41598-018-33582-w

**Published:** 2018-10-18

**Authors:** Jackelyn M. Kembro, Sonia Cortassa, David Lloyd, Steven J. Sollott, Miguel A. Aon

**Affiliations:** 1 Instituto de Investigaciones Biológicas y Tecnológicas (IIByT-CONICET), and Instituto de Ciencia y Tecnología de los Alimentos, Cátedra de Química Biológica, Facultad de Ciencias Exactas, Físicas y Naturales, Universidad Nacional de Córdoba, Velez Sarsfield 1611, Córdoba, X5000HUA Cordoba Argentina; 2 Laboratory of Cardiovascular Science, National Institute on Aging, NIH. 251 Bayview Boulevard, Baltimore, 21224 MD USA; 3 School of Biosciences, Cardiff University, Main Building, Museum Avenue, Cardiff, CF10 3AT Wales UK

## Abstract

Mitochondria serve multiple key cellular functions, including energy generation, redox balance, and regulation of apoptotic cell death, thus making a major impact on healthy and diseased states. Increasingly recognized is that biological network stability/instability can play critical roles in determining health and disease. We report for the first-time mitochondrial chaotic dynamics, characterizing the conditions leading from stability to chaos in this organelle. Using an experimentally validated computational model of mitochondrial function, we show that complex oscillatory dynamics in key metabolic variables, arising at the “edge” between fully functional and pathological behavior, sets the stage for chaos. Under these conditions, a mild, regular sinusoidal redox forcing perturbation triggers chaotic dynamics with main signature traits such as sensitivity to initial conditions, positive Lyapunov exponents, and strange attractors. At the “edge” mitochondrial chaos is exquisitely sensitive to the antioxidant capacity of matrix Mn superoxide dismutase as well as to the amplitude and frequency of the redox perturbation. These results have potential implications both for mitochondrial signaling determining health maintenance, and pathological transformation, including abnormal cardiac rhythms.

## Introduction

Chaos can be defined as a dynamic, non-periodic behavior which appears random, exhibiting dependence on initial conditions, and it arises in deterministic nonlinear systems under certain conditions. Chaos is qualitatively different from periodic behavior, not only because of its erratic appearance, but also due to its sensitivity to small perturbations of initial conditions^1–3^. Although unstable, chaotic dynamics is also bounded, as revealed by the multiple trajectories present in a *strange attractor*, a restricted region of the phase space to which all the trajectories settle or approach over time^4^. Biological systems are not strictly deterministic because of noise, which can be both intrinsic and extrinsic to the system^5^.

The ubiquitous presence of chaos in nature has been ascertained in a plethora of phenomena (see^4^ for a review) ranging from enzyme-catalyzed reactions^6,7^, metabolic pathways^8,9^, yeast metabolism^10,11^ to the food web of the plankton community^12^, as well as neuronal activity^13^ and cardiac rhythms^14–18^. Notwithstanding, the *biological significance* of chaos is still unclear. In physiology two apparently opposing roles have been assigned to chaos – either implying pathological conditions, “dynamical diseases”^1^, or that health is necessarily characterized by highly irregular and complex dynamics^19^ that bestow robustness and stability to biological networks, yet enabling adaptability under environmental pressure.

As a potentially lethal ventricular rhythm, cardiac fibrillation is considered a form of spatiotemporal chaos^15^ arising from perturbations in electrophysiological behavior related to early after-depolarizations^20^ or alternans^17,21,22^. Synchronized mitochondrial oscillations in membrane potential (ΔΨ_m_), NADH, and ROS have been observed *in vitro* and in heart-reperfusion-after ischemic injury, producing cellular inexcitability while setting the stage for cardiac fibrillation^23,24^. However, completely unknown is whether mitochondria can exhibit chaotic behavior and if this type of dynamics can be at the origin of ventricular fibrillation.

Deterministic mathematical models of a biological system constitute a valuable tool for exploring chaos; potentially, they can reveal intrinsic dynamic properties of complex systems, while not being contaminated by noise. In this context, the concerted interplay between superoxide dismutase (SOD) activities in both mitochondrial and extra-mitochondrial compartments determines the degree of redox imbalance, a decisive trait for the appearance of mitochondrial oscillations, according to a deterministic, bi-compartmental Mitochondrial Energy-Redox (ME-R) model^25^. In this experimentally validated computational model, mitochondrial dynamic behavior along a specific “edge” (i.e. the boundary between stability-instability, or functional-dysfunctional) region of the parametric space can exhibit complex oscillatory behavior. This “edge” delimits fully functional from pathological mitochondrial function^26^.

In this work, we seek to demonstrate that mitochondria can behave chaotically, and to delineate the conditions leading to chaos, and its characterization. We hypothesize that unstable complex oscillatory dynamics sets the stage for the appearance of chaos. The results show that, at the “edge”, chaotic behavior in mitochondrial function can be triggered by even a mild, regular sinusoidal redox forcing perturbation. We discuss the possible implications of our findings for both health and disease.

## Results

### From complex to chaotic oscillatory behavior at the “edge” of stability

**Figure 1:**
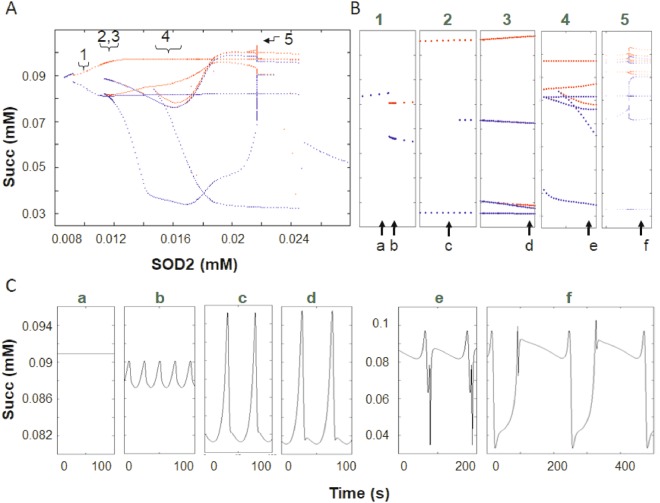
Bifurcation diagram of the TCA cycle intermediate Succinate (Succ) as a function of Mn superoxide dismutase (SOD2) in the absence of an externally forced perturbation. Bifurcation analysis of succinate dynamics as a function of matrix SOD2 concentration reveals complex bifurcation properties. (**A**,**B**) Transitions from a fixed point to limit cycle, and subsequent period doublings can be clearly observed, followed by a return to a fixed point for larger SOD2 concentrations. Maxima and minima values of the steady state oscillations are represented by red and blue dots, respectively. Brackets and arrows in A indicate the parametric region zoomed in and depicted in the corresponding panels in (**B**). In (**B**) arrows and letters (a-f) indicate the parameter value corresponding to the time series depicted in (**C**). Notice that complex waveform changes were concomitant with the bifurcations in dynamic behavior. (**C**) In the absence of external superoxide perturbation (amplitude and period = 0), the concentration values of SOD2 (in mM) used in the simulations and displayed in panels a-f were: 0.0092, 0.0092272, 0.0112851, 0.0145, 0.016 and 0.02167268014, respectively (see arrows and letters in **B**) with SOD1 = 9.7 10^−5^) under all conditions. Shunt was set at 0.04. See also attractor reconstruction for time series “f” in Supplementary Fig. S2.

To investigate the conditions leading to the triggering of chaotic dynamics, we first evaluated the dynamics of the model without external perturbations. Figure 1 shows the bifurcation diagram of succinate (Succ), an intermediate of the TCA cycle in the mitochondrial matrix, as a function of SOD2 (Supplementary Fig. S1). Within a narrow range of variation in SOD2 concentration (~10 to 25 μM), Succ levels display wide excursions (from ~30 to 100 μM). As SOD2 concentration increases the system’s dynamics transitions from a fixed-point attractor through multiple and successive period doublings (Fig. 1B) accompanied by complex changes in the oscillations’ waveform and period (Fig. 1C), which are then followed by a return to a stable fixed-point for SOD2 concentrations >24.5 μM. Moreover, complex Succ oscillations were observed with up to 16 peak values (Fig. 1C, panel f, power spectrum in Supplementary Fig. S2A, phase space reconstruction Supplementary Fig. S2D). At this complex oscillatory behavior, Succ exhibits an intricate relationship with other mitochondrial variables (Supplementary Fig. S2E). Although the appearance of complex oscillatory behavior is characterized by the existence of period doublings, we were unable to find chaos, even after thoroughly examining the parametric space. Comparable results were obtained in the bifurcation diagram of SOD1 and Shunt (i.e., fraction of electrons from respiration diverging toward ${{\rm{O}}}_{2}^{\cdot -}$) (Supplementary Fig. S5). As previously described^26^, the realm of complex oscillations (without chaos) was located at the edge between normal (high antioxidant capacity, thus low ROS generation) and pathological mitochondrial energetic behavior (low antioxidant capacity, high ROS generation) (Supplementary Fig. S9).

**Figure 2:**
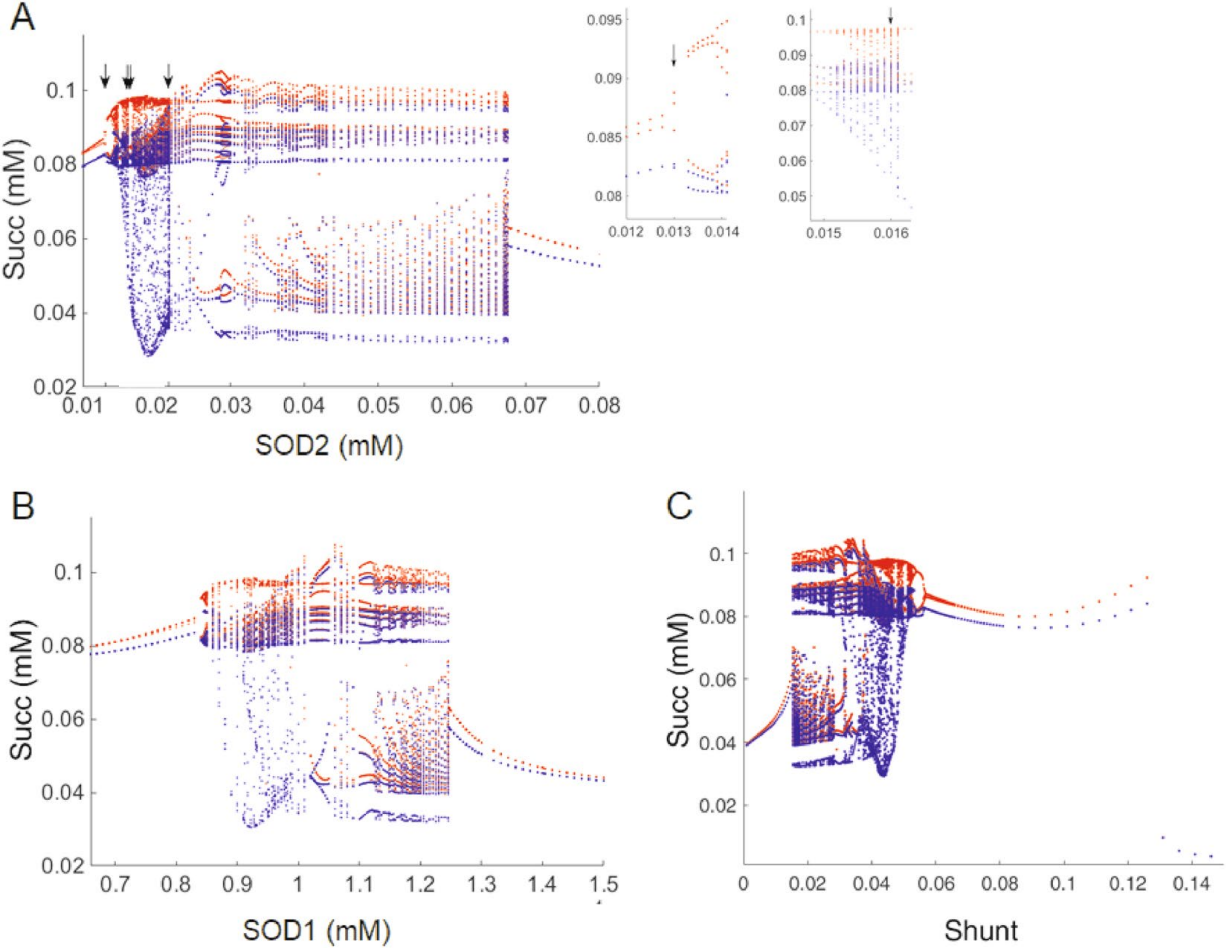
Exogenous forcing oscillatory superoxide perturbation elicits chaotic dynamics. Bifurcation diagram corresponding to the concentration of the TCA cycle intermediate Succinate (Succ) as a function of the parameters **A**) matrix Mn superoxide dismutase (SOD2) concentration (at constant 9.7 10^−5^ mM SOD1 concentration and Shunt 0.04), (**B**) extra-mitochondrial Cu, Zn superoxide dismutase (SOD1) concentration (at constant mitochondrial SOD2 = 0.021679 and Shunt 0.04) and (**C**) the fraction of redox electrons from respiration diverging toward O_2_^−^ (“Shunt”) at (SOD1 = 9.7 10^−5^ mM and SOD2 = 0.021679) in the presence of externally forced oscillatory superoxide perturbation. In inset (**A**) magnification of two SOD2 parametric regions are shown. Notice how limit cycles are followed by successive period doublings leading to chaotic dynamics. Maxima and minima values of steady state oscillations are represented in red and blue, respectively. Arrows point to the concentrations of SOD2 used in Figs 3, 4, 6 and Supplementary S3. External superoxide perturbation: amplitude = 10^−7^ mM, period = 30 s.

At this edge region, where the system oscillates autonomously (i.e., without exogenous forcing), injecting an exogenous sinusoidal perturbation in extra-mitochondrial superoxide (${{\rm{O}}}_{2}^{\cdot -}$_i_, 100 pM amplitude, 30 s period) elicits both complex oscillatory and chaotic dynamics. Figure 2 depicts the bifurcation diagram of Succ levels, using the concentration of SOD2 (Fig. 2A) or SOD1 (Fig. 2B) or ROS generation in the respiratory chain (“Shunt”; Fig. 2C), as bifurcation parameters. Notice the increase in complexity in the bifurcation properties of the Succ dynamics leading to chaos (inset Fig. 2A) as compared to Fig. 1B. Interestingly, the addition of this perturbation not only triggers the appearance of a chaotic regime, but also extends the range of SOD2 concentrations in which oscillatory/chaotic dynamics become apparent (range ~15 to 70 μM SOD2 (Fig. 2A) *vs*. ~8 to 24.5 μM (Fig. 1A)). Inspection of other variables such as mitochondrial H_2_O_2_ (Supplementary Fig. S6A) or extra-mitochondrial GSH (Supplementary Fig. S6B) provides evidence of differential sensitivity to SOD2 depending on the variable analyzed. For example, Succ (Fig. 2A) and H_2_O_2_ (Supplementary Fig. S6A) show complex dynamic behavior as a function of SOD2 > 30 μM, whereas at these concentrations GSH exhibited only smooth oscillations (Supplementary Fig. S6B).

### Characterization of chaotic dynamics in mitochondrial function

**Figure 3:**
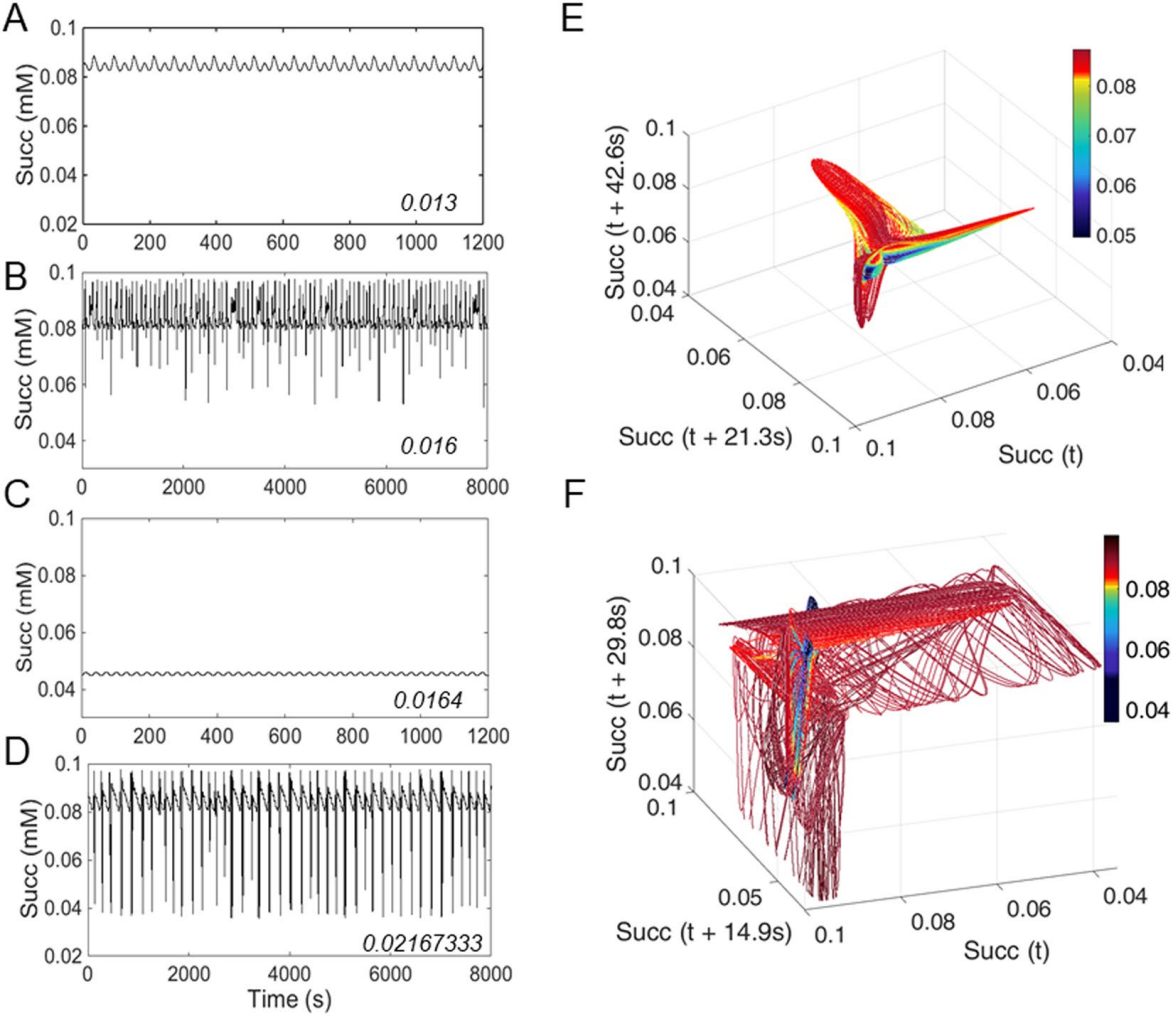
Sensitivity of chaotic attractor dynamics to changes in mitochondrial antioxidant capacity. The sensitivity of chaotic behavior was investigated as a function of changes in matrix SOD2 concentration. Succinate time series performed for SOD2 concentrations (in mM) 0.013 (**A**); 0.016 (**B**); 0.0164 (**C**) and 0.0216733 (**D**), as indicated by arrows in Figure 2A. (**E**,**F**) Reconstructed attractor performed for SOD2 concentrations (in mM) 0.016 (E, corresponding to time series in **B**) and 0.02167 (**F**, corresponding to time series in **D**). Color coding represents a fourth time lag Succ (t + 63.9 s) and Succ (t + 44.7 s), respectively. Model-simulated time series were calculated with Shunt = 0.04, SOD1 9.7 10^−5^ mM. External superoxide perturbation: amplitude = 1 10^−7^ mM, period = 30 s. For details on estimation of the time lag and embedding dimension refer to Fig. S3. Note that these 3D space attractors are not completely unfolded. See Supplementary Figure S4 for attractor reconstruction corresponding to oscillatory time series obtained at SOD2 concentrations 0.013 and 0.0164 mM.

At the “edge”, given the relevant role played by SODs and ROS generation in the respiratory chain (“Shunt”) in delimiting stable from unstable states in mitochondrial function, in both cytoplasmic and mitochondrial compartments, we sought to further investigate their impact on the appearance of chaotic dynamics. The bifurcation diagrams of the three mitochondrial processes involved in regulating superoxide concentration, namely SOD2 (Fig. 2A), SOD1 (Fig. 2B) or Shunt, (Fig. 2C), indicate that regulation of O_2_^.−^ concentration and compartmentation are at the heart of the control of mitochondrial dynamics under these conditions. As a result, slight parametric changes from either of the three processes lead to drastic qualitative changes in dynamics. For example, Succ time series display smooth low amplitude oscillations at 13 μM (Fig. 3A, Supplementary S4A) and 16.4 μM SOD2 (Fig. 3C, Supplementary S4C), whereas at 16 μM and 21.67333 μM SOD2 (Fig. 3B,D), complex chaotic dynamics are observed.

Phase space reconstruction is a valuable tool for visualizing the underlying properties of mitochondrial dynamics (Supplementary Figs S2, S4)^4,27^. Phase space plots of representative time series (Fig. 3A–D) are shown in Supplementary Figures S4A, 3E, S4C and 3F, respectively. As a caveat, the attractors described by Succ phase portraits are not completely unfolded at 3D representation (Fig. 3E,F), because the embedding dimensions of these attractors are approximately 4 and 13, respectively. The attractors shown provide additional evidence that mitochondrial dynamic behavior exhibits high sensitivity to the antioxidant capacity of its matrix via SOD2, switching back and forth between oscillatory and chaotic regimens, as indicated by limit cycle (Supplementary Fig. S4A,C) or strange attractor (Fig. 3E,F) behavior in phase space, respectively, depending on the SOD2 matrix concentration levels.

**Figure 4:**
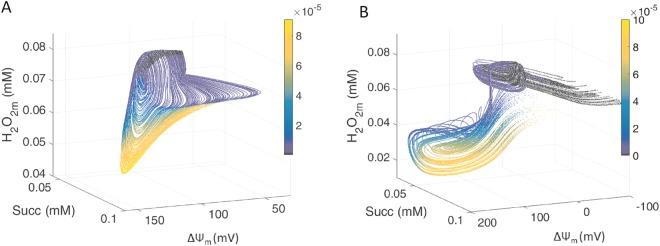
The dynamic relationship between mitochondrial ROS and energetics is sensitive to changes in mitochondrial antioxidant capacity. Phase space plots were performed for SOD2 concentrations (in mM) 0.016 (**A**); and 0.0216733 (**B**). Color coding represents extra-mitochondrial superoxide concentration. Model-simulated time series were calculated with Shunt = 0.04, SOD1 9.7 10^−5^ mM. External superoxide perturbation: Amplitude = 1 10^−7^ mM, period = 30 s. Key to symbols: Succ, succinate, ΔΨ_m_, membrane potential, H_2_O_2_m, matrix hydrogen peroxide.

In deterministic systems, chaos has been defined as non-periodic behavior, with both bounded and unstable dynamics, which are represented by *strange attractors*, a signature of chaos^2,5,28^; (reviewed in^4^). In this context note that in Fig. 3E,F all trajectories are limited to a restricted region of state space as time evolves, whereupon they flow in a deterministic but complex and unpredictable manner^29^. Phase space representation of Succ *vs*. major energetic (mitochondrial membrane potential, ΔΨ_m_) and redox (matrix hydrogen peroxide, H_2_O_2_m) variables are displayed in Figure 4 and Supplementary Figure S4. Interestingly, the association between these critical variables also depends on SOD2 concentration, which dictates the dynamics of mitochondrial function, as can be judged from the eliciting of chaotic dynamics by two different SOD2 concentrations with distinct relationships between variables.

**Figure 5:**
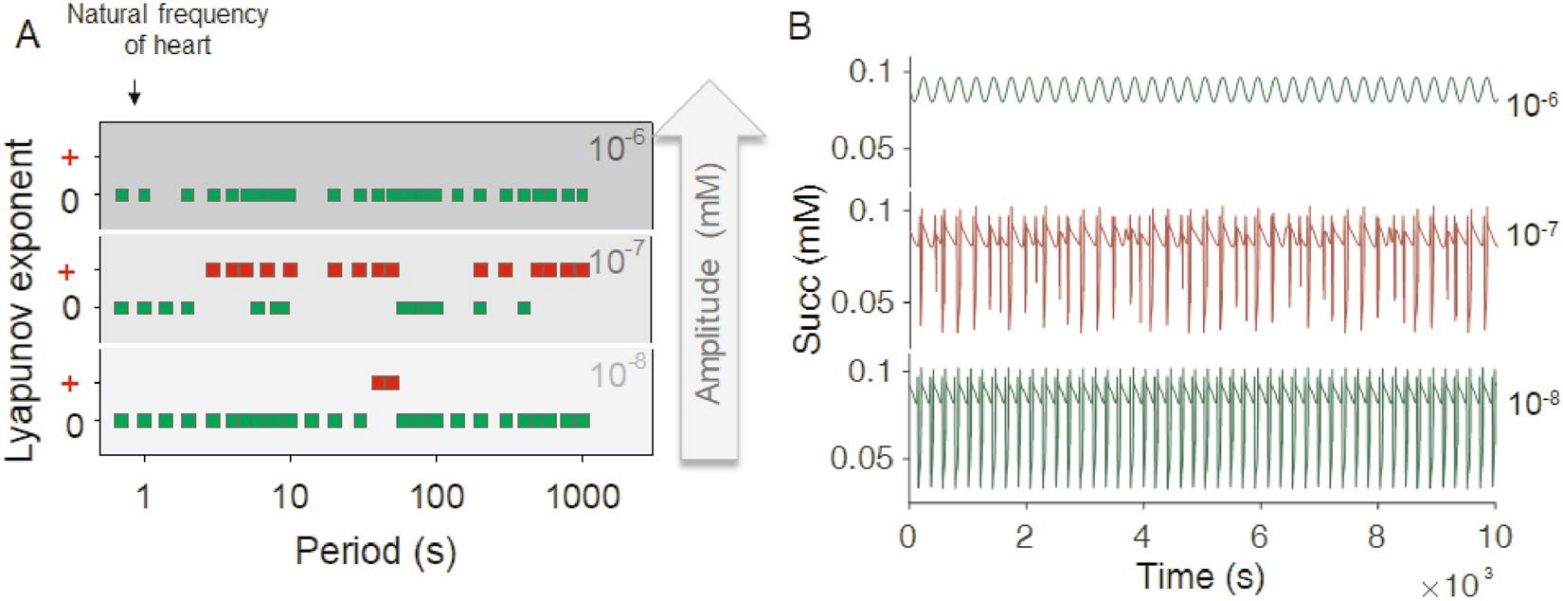
Sensitivity of chaotic behavior to parameters of the redox perturbation. (**A**) A color-coded matrix of dominant Lyapunov exponents was constructed as a function of amplitude (*a*) and period (ω = 2 π (1/Period)) of the superoxide perturbation (Methods, equation 1). Red squares indicate positive dominant Lyapunov exponents, a signature of chaos, while green squares denote Lyapunov exponents = 0, thus not corresponding to chaos. (**B**) Depicted are time series estimated for a 200 s period. Dark green (amplitudes 10^−6^ and 10^−8^ mM) and dark red (amplitude 10^−7^ mM) time series show zero and positive dominant Lyapunov exponents, respectively. Note entrainment at the highest amplitude of 10^−6^ mM while complex oscillations are observed for the lowest amplitude 10^−8^ mM. Model-simulated time series were calculated with SOD2 = 0.0216733 mM, SOD1 = 9.7 10^−5^ mM, and Shunt = 0.04.

The Lyapunov exponent, a main signature indicator of chaos that quantitates the average rate of convergence or divergence between neighboring trajectories in phase space, was positive as expected for chaotic dynamics^11,30^. Both the properties of bounded dynamics (within an attractor) and sensitive dependence to initial conditions (as determined by the dominant positive value 0.025 of the Lyapunov coefficients for 21.67333 μM SOD2), are present in the chaotic behavior exhibited by mitochondrial function. Power Spectral Analysis of these time series showed a more densely populated spectrum of relevant frequencies at 21.67333 μM (Supplementary Fig. S3D) compared to 16 μM SOD2 (Supplementary Fig. S3C). A visual matrix representation of chaotic behavior based on Lyapunov exponents is depicted in Fig. 5, as a function of the amplitude and period of the forcing sinusoidal function describing the superoxide perturbation. The matrix plot enables the realization that chaos only occurs under selective amplitudes and periods of the perturbation. Interestingly, a detailed evaluation of the time series obtained at forcing amplitudes of 10^−8^ mM showed that the dynamic behavior of the system is like that of the autonomous system without forcing (Fig. 5B). Consequently, the dynamic response of the forced ME-R model becomes the same, or very similar, to that of the forcing function, indicating the presence of entrainment, i.e., the period of the oscillatory response is an integer multiple of the forcing period^31^. Moreover, during entrainment all state variables of the model display oscillations equal to the forcing period and with similar power spectra (Supplementary Fig. S8). For forcing amplitudes of 10^−5^ mM at periods comprised between 1 sec up to 1000 sec, model simulations showed that mitochondrial function is impaired, i.e., mitochondria oscillate around values of ΔΨ_m_ ~ 0 and NADH ~ 99% oxidized (Supplementary Fig. S10).

**Figure 6:**
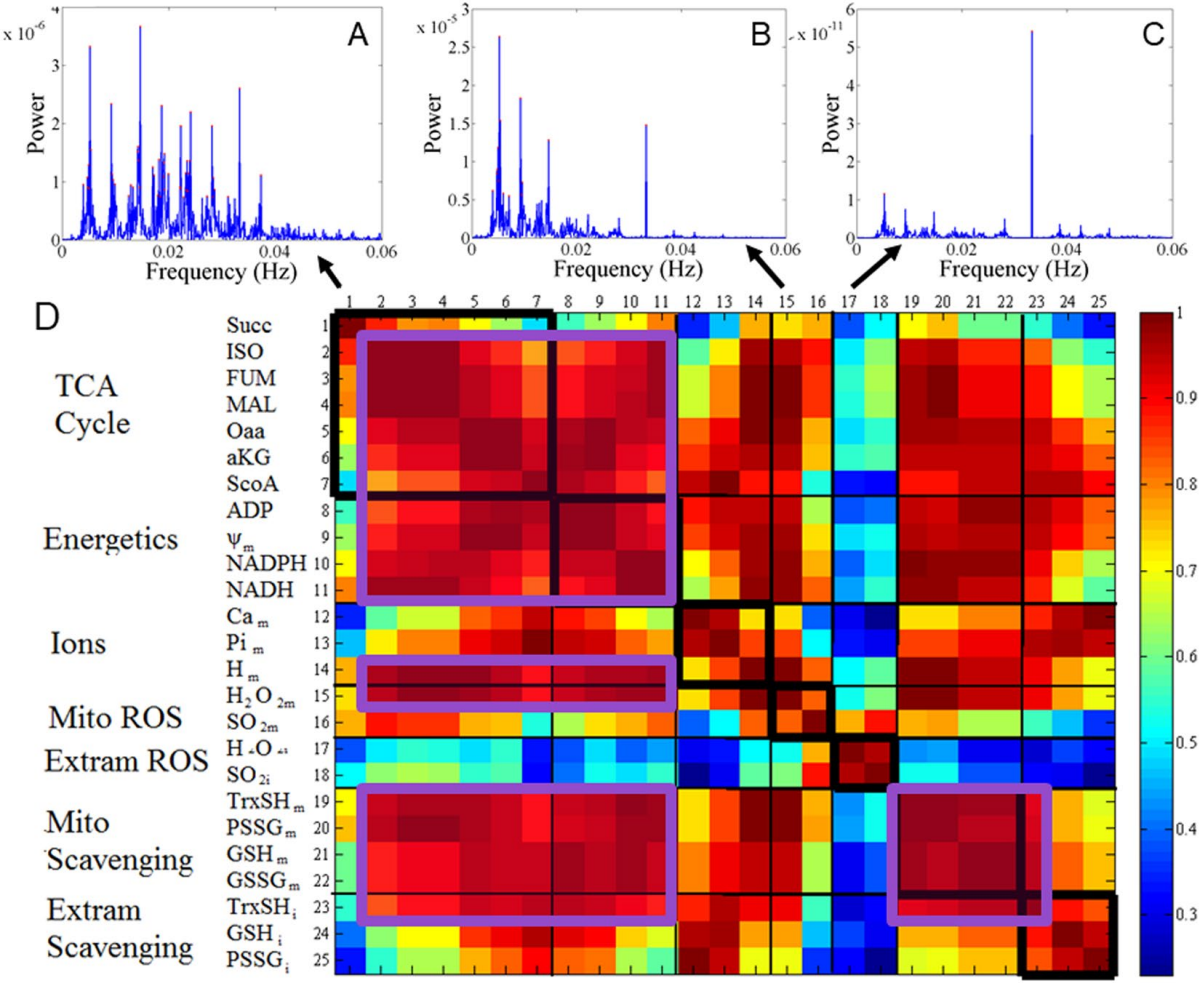
Temporal synchronization between oscillatory variables during chaotic dynamics. Synchronization between oscillations in state variables of mitochondrial function within the chaotic regimen was analyzed through pair-wise estimation of the correlation between periodograms of each variable as obtained with Power Spectrum Analysis (PSA). Displayed are the PSA of (**A**) succinate, (**B**) mitochondrial H_2_O_2_, and (**C**) extra-mitochondrial superoxide (SO_2_m). (**D**) Correlation matrix obtained from the power spectra of model variables. Strong correlations between variables (shown in red) indicate main shared frequencies. Note that Succ shows intermediate and low levels of correlations with other variables, given the multiple principal frequencies detected by PSA (panel A). A low level of correlation between extra-mitochondrial superoxide and H_2_O_2_ (represented in blue) and the rest of the variables is observed given that only one predominant frequency is shared between these ROS variables while four or more were exhibited by most variables. Black boxes: functional grouping of variables into 6 categories: tricarboxylic acid (TCA) cycle, Energetics, Ions, Mitochondrial ROS, Extra-mitochondrial ROS, Mitochondrial and Extra-mitochondrial ROS scavenging systems. Magenta boxes: grouped variables by frequency similarity according to PSA and large correlation coefficients (orange-red).

Together, the several lines of evidence presented support the idea that the irregular dynamics exhibited by mitochondrial function at the “edge”, in the presence of external redox perturbation, corresponds to chaos. The exquisite sensitivity of mitochondrial dynamic “motion” (e.g., ΔΨ_m_ and redox trajectories) at the edge of stability agrees with the thesis that redox perturbations under oxidative stress play a leading role in the triggering of chaotic dynamics.

### The dynamic architecture of chaotic mitochondrial function

The multiple frequencies and amplitudes characterizing the complex oscillations exhibited by mitochondrial dynamics at the “edge” are linked via an inverse power law relation^26^, which is a signature of fluctuations occurring over widely different time scales (inset Supplementary Fig. S3B,D). However, dominant principal frequencies are also apparent even when a time series is chaotic with a frequency equal or as a multiple of the forcing frequency (0.0333 Hz corresponding to the 30 s period). Hence, we assessed the functional interdependence between mitochondrial processes *within* the chaotic regimen, utilizing pair-wise correlation between periodograms of each state variable in the model.

Figure 6 depicts the correlation matrix of the principal shared frequencies between mitochondrial processes, as revealed by PSA. Correlation analysis between variables (strong correlation displayed in red) indicates that Succ (Fig. 6D, first column) shows intermediate and low levels of correlation with other variables (in yellow and blue, respectively), given the multiple principal frequencies exhibited by its power spectrum (Fig. 6A). Amplitude- delay plots showed that Succ amplitudes can range from ~3 to 60 μM during chaotic dynamics (Fig. S7 blue circles). The color-coded matrix (Fig. 6) reveals strong correlations between oscillators corresponding to state variables from the TCA cycle (except Succ), OxPhos, pH, mitochondrial ROS species (H_2_O_2_, O_2_^.−^) and mitochondrial ROS scavenging as marked by magenta boxes in Fig. 6D; this result underscores the functional interdependence between energetics and ROS scavenging. In contrast, a low level of correlation (denoted in blue) between extra-mitochondrial O_2_^.−^ and H_2_O_2_ and the remaining variables (except mitochondrial O_2_^.−^) is observed given that only one predominant frequency is observed in these ROS species (coinciding with the period of the imposed O_2_^.−^ perturbation) while four or more shared spectral peaks were exhibited by most variables.

## Discussion

A main contribution of the present work is to show that, under oxidative stress, resulting from the interplay between SODs compartmentation and ROS generation in the respiratory chain, mitochondrial function can exhibit chaotic dynamics. Under these conditions, we demonstrate that a regular sinusoidal redox perturbation can trigger chaotic dynamics in a high dimensional deterministic system, represented by a bi-compartmental computational model of mitochondrial function while exhibiting unstable complex oscillatory behavior. Applying bifurcation analysis, time delay plots, phase space attractor reconstruction, and calculation of Lyapunov exponents^27^, we demonstrate that the irregular mitochondrial dynamic behavior resulting from perturbation at the “edge” exhibits the expected signatures of chaos. These are sensitively dependent on initial conditions as indicated by positive dominant Lyapunov exponents (Fig. 5), and strange attractors (Fig. 3E,F), the latter as also revealed by 3D phase portraits of main representative energetic (ΔΨ_m_), metabolites (Succ) and redox (H_2_O_2_m) state variables (Fig. 4).

Complex oscillatory behavior^26^ as well as onset of chaotic dynamics in the ME-R model are shown to be dependent upon the interplay between SOD1 and SOD2 and ROS generation in the respiratory chain (Figs 2 and 3). At amplitudes 10^−7^ mM the external forcing O_2_^.−^ oscillation triggers chaos over a broad range of periods (4 to 1000 s) (Fig. 5). Amplitudes of 10^−6^ mM lead to entrainment where simple oscillations can be observed with the principal frequency equal to that of the external forcing O_2_^.−^ perturbation (Supplementary Fig. S8). Forced-autonomous oscillators can be driven to chaos or entrainment^32^ depending on the frequency and amplitude of the perturbation, with periods that are integer multiples of the forcing period^33,34^.

The likelihood that mitochondrial chaos could potentially be involved in cardiac fibrillation has been anticipated by previous reports showing that mitochondrial ΔΨ_m_ oscillations drive cardiac myocyte inexcitability^35^, which in the myocardial syncytium can generate irregular electrophysiological dynamics and fibrillation^23,36–38^. Impairment of mitochondrial function after ischemic injury generates ROS-elicited synchronized mitochondrial oscillations^35,39,40^, leading to cardiomyocyte and heart dysfunction while setting the stage for catastrophic arrhythmias^23,41,42^. Although cardiac fibrillation has been associated with chaotic electrophysiological dynamics^15^, it is completely unknown whether chaotic energy-redox behavior of mitochondrial origin can elicit a wider spectrum of cardiac arrhythmias. A possible origin of the redox perturbation leading to chaos in our model simulations can be given by the heart rhythm itself, since it has been shown that heart mitochondria exhibit oscillatory rates of ROS generation^43^ in response to transitions from high to low energy demand as it happens during the normal systolic-diastolic cycle. In this regard, chaos can appear at periods (≥3.2 s = 19 bpm, at 10^−7^ mM amplitude), i.e. longer than the frequency of the human heart at rest (1 Hz = 1 beat/s = 60 bpm [beats/min]) (Fig. 5). Additionally, at perturbation periods within the range of the normal heart rate 1–0.6 s (60 to 100 bpm), as well as lower periods (0.2, 0.24, 0.26 or 0.3, equivalent to 300, 250, 225 or 200 bpm, at 10^−7^ mM amplitude) associated with tachycardia (data not shown) only periodic dynamics are observed, independently from the amplitude of the forcing external O_2_^.−^ oscillation. Overall, our findings predict that in the heart the occurrence of chaos could happen only under pathological heart rate conditions, consistent with clinical evidence.

Mitochondrial dysfunction leads to energy depletion and electrical instability in the heart increasing the vulnerability to the initiation of arrhythmias. Previous evidence strongly supports the hypothesis that mitochondrial ΔΨ_m_ instability and GSH redox recovery play major roles in post-ischemic arrhythmias while suggesting that mitochondria are key potential targets for antiarrhythmic interventions^36,37,42,44^. Rapid activation of ATP-sensitive K^+^ (K_ATP_) channels on energy depletion cause action potential duration (APD) shortening^35,38,45,46^. Oxidative stress drives the state of the mitochondrial network to *criticality*^39,47^, a situation in which even small perturbations will trigger cell-wide depolarization in the form of a propagated mitochondrial membrane potential (ΔΨ_m_) depolarization wave followed by sustained, self-organized, low frequency, high amplitude oscillations in ΔΨ_m_^35,48^. The rapid ΔΨ_m_ depolarization transforms mitochondria from generators into consumers of ATP, causing a drop in the cellular ATP/ADP ratio, activating K_ATP_ channels, and shortening the APD^35,42,45,46^. Energetic collapse resulting from the formation of a metabolic sink induced by regional mitochondrial depolarization^42^, deeply affects myocardial electrical propagation via decrease of the action potential amplitude and duration, reducing wavelength while introducing regions of short refractory period that facilitate re-entry^38^.

Temporal correlations are a signature of functional interdependence which can occur across a wide range of time scales^49^, or as shown in this work among oscillators from mitochondrial processes operating within the same time scale (seconds) while exhibiting multiple alike frequencies (Fig. 6). Multi-oscillatory frequencies on different time scales, from minutes to several hours, appear to be embedded in a chaotic attractor^50^, of which mitochondria and other components of the redox balancing machinery take part. At the functional “edge” (Supplementary Fig. S9), a mitochondrion displaying chaotic dynamics could be sensed by the cell via its emitted ROS signaling, and appropriately tagged for repair or recycling through mitophagy^51,52^ depending upon the characteristics of chaos as revealed by strange attractors (Figs. 3 and 4). Consequently, the fact that mitochondrial H_2_O_2_ displays chaotic dynamics becomes crucial since this ROS species that can be a signaling molecule^53,54^ may trigger mitophagy as well as act as a potent inducer of cellular senescence^55^.

It is also well known that mitochondrial ΔΨ_m_ is a key health reporter of this organelle, with dedicated “tag” proteins such as PINK1 and Parkin^56^, the former being readily detected in damaged mitochondria after depolarization^57^. We propose that the complex oscillatory dynamics shown here, when mitochondrial function is on the “pathophysiological edge”, could also function as frequency-amplitude encoded ΔΨ_m_ signals (Fig. 4, comparing ΔΨ_m_ dynamics between panels A and B). The resulting strength of the molecular attraction (encoded in the patterns of depolarization) of PINK1 to mitochondria integrating the ΔΨ_m_ signal, serves as a general “go/no-go” signal to propagate an appropriately timed activation of the mitophagy cascade.

In summary, we report for the first-time chaotic dynamics in an extensively validated computational model of mitochondrial function. Chaos can be triggered by a regular, sinusoidal redox perturbation in a parametric region where mitochondrial function also exhibits entrainment and unstable complex oscillatory dynamics, whereby it becomes exquisitely, and exclusively, sensitive to the interplay between SOD1 and SOD2 and the balance of ROS production and scavenging within intra- and extra-mitochondrial compartments. Consequently, a major prediction from this work would be that, under oxidative stressful conditions, titrating the levels of SOD2 would not only sensitively change mitochondrial dynamics and the characteristics of the chaotic attractor within the “edge” domains (Supplementary Fig. S9), thereby shifting between limit cycle and chaotic dynamic regimens (Figs 3 and 4), but may also, depending upon SOD2 level, drive mitochondrial function away from the “edge” toward stable or pathological function.

## Methods

### Model description

The ME-R model accounts for energetic-redox, ionic processes, pH regulation, and their interactions as well as transport between compartments^25,26^ (Supplementary Fig. S1).

A natural oscillator subject to periodic forcing with adjustable frequency and amplitude may behave non-periodically or be entrained to oscillate with an integer multiple of the forcing period^58^. Periodically forced low dimensional systems (e.g., two degrees of freedom) can show three possible forms of behavior: periodic, quasiperiodic and chaotic^31^. Although ME-R is a complex, high dimensional, model (Supplementary Fig. S1), it has been shown that the principal oscillator is a subsystem, comprising antioxidant enzymes and ROS generation, directly associated with IMAC^48^. IMAC transports O_2_^.−^ and its conductance is modulated via a negative feedback loop by extramitochondrial O_2_^.−^ concentration^48^. Thus, to perturb mitochondrial dynamics in our model simulations, we employed a forcing sinusoidal fluctuation of O_2_^.−^ in the extra-matrix compartment, as follows:

1 $$d{[{{\rm{O}}}_{2}^{-}]}_{i}/dt=({{\rm{v}}}_{{\rm{m}}}{/v}_{{\rm{i}}})\,{{{\rm{V}}}^{{\rm{Tr}}}}_{{\rm{ROS}}}-{{\rm{V}}}_{{\rm{CuZnSOD}}}+a\,\sin \,{\rm{\omega }}\,t+a$$

where the parameters *a* and ω, represent the amplitude and period (ω = 2 π (1/Period)). The first two terms are the same in the original ME-R model^25^, hence *a* = 0 becomes the non-forced situation. The last term is necessary to avoid negative O_2_^.−^ values. The periods utilized ranged from 0.2 to 1000 s and amplitudes from 10^−6^ to 10^−8^ mM, a physiologically realistic range according to previous evidence^25,26^.

### Analytical methods

Numerical integration of the ME-R model equations was performed with MatCont 2.4^59^ in MATLAB 7.1, until steady-state solutions were obtained (i.e., when the magnitude of each time derivative was <10^−10^). Time series with duration of at least 6 × 10^6^ ms were constructed by numerical integration of model equations. To allow transient states to vanish, the system was computed during a time lapse of 2 × 10^8^ ms. The solutions were then evaluated with the function deval.m^60^ in MATLAB R2017a to obtain a time series with constant sampling frequency at 1 ms. All studies were performed using the parameter setting optimized in our previous work^25,26^, with ADPm = 0.01 mM, i.e. consistent with energized mitochondria under state 4 respiration.

***Bifurcation diagrams:*** also called orbit diagrams, constitute a graphical representation of all possible steady state values of a variable as a function of a control (or bifurcation) parameter. These diagrams can provide evidence of bifurcations (i.e., qualitative changes in the dynamic behavior of a system, their stability, or the topological structure of its phase portrait as one or more parameters pass through a critical value)^29^. For simplicity, we have represented only the peak and valley values from time series showing oscillatory behavior (e.g., a sinusoidal oscillation is represented by two points (one for each maximum and minimum value, respectively). In the diagram, a bifurcation in a branch of steady states indicates period doubling.

***Phase space reconstruction*** was performed for the discretely sampled time series of the state variables from the ME-RM model. For *phase space reconstruction*, y(t) = [x(t), x(t + τ), x(t + 2τ)…], the time lag (τ) value was determined from the first minimum of the non-linear correlation function called *average mutual information*, and was computed using the open access MutualInfo 0.9 package^61,62^ in MATLAB R2017a. The appropriate *embedding dimension* was calculated according to the false-nearest neighbor technique to determine the number of dimensions needed for the complete unfolding of the geometrical structure of the attractor (i.e., points should lay close to one another in the phase space due to their dynamics but not their projection)^27^, using open access code^63^ in MATLAB R2017a. For additional information see Supplementary Information SI1.

#### Lyapunov exponents

These exponents quantify the average rate of convergence or divergence of two neighboring trajectories in phase space^27^, and serve as a measure of the sensitivity to initial conditions^64^. Since Lyapunov exponents are derived from the average of local divergences and/or convergences from many trajectories over the entire attractor, they quantify global rather than local behavior. Any system containing at least one positive Lyapunov exponent is defined as being chaotic, with the magnitude of the exponent reflecting the time scale on which the system dynamics becomes unpredictable^65^. Hence estimation of the dominant (largest, most positive) exponent is especially important^64^. Herein, the dominant Lyapunov exponent was estimated with the open access program FET (and its preprocessor BASGEN) in MATLAB R2017a based on the widely used methodology first described in^64^. FET/BASGEN makes use of the method of phase space reconstruction and obtains the long time average rate of divergence of nearby orbits by averaging the local rates of orbital divergence divided by the total travel time along the orbit.

### Power Spectrum Analysis

(PSA, also called Fourier analysis) is a method in which a periodogram is constructed; if there is periodic oscillatory behavior in the data set, its period will appear as a peak in the spectral energy. We used the Fast Fourier Transform (FFT) subroutine of MATLAB R2017a to perform PSA on the time series. To assess potential correlations in the temporal dynamics of mitochondrial variables, pair-wise correlations between the periodograms of all variables were performed. A high correlation implies similarities in the periodogram, meaning that the state variables share the same principal oscillatory frequencies, whereas a correlation value of 0 indicates that both time series present completely different principal oscillatory frequencies.

### Code availability

The ME-R model is available upon request, and state variable initial conditions for simulations can be found in Supplementary Table S1.

## Electronic supplementary material

Supplementary Information

## Data Availability

All data generated or analyzed during this study are included in this published article (and its supplementary information files). Data time series, depicted in Figure 3, with parameter settings SOD2 concentrations (in mM) 0.013; 0.016; 0.0164 or 0.0216733, Shunt = 0.04, SOD1 9.7 10^−5^ mM. External superoxide perturbation amplitude = 1 10^−7^ mM, period = 30s, are publicly available on figshare^66–69^.
