# Supplementary material for: Mitochondrial chaotic dynamics: Redox-energetic behavior at the edge of stability
Source: Sci Rep. 2018 Oct 18;8:15422. doi: 10.1038/s41598-018-33582-w (PMC6194025; doi:10.1038/s41598-018-33582-w)
Supplement: Supplementary file 1 — Supplementary Information [file 41598_2018_33582_MOESM1_ESM.pdf]

Mitochondrial chaotic dynamics: Redox-energetic behavior at the edge of stability

Jackelyn M. Kembro, Sonia Cortassa, David Lloyd, Steven J. Sollott, Miguel A. Aon*

Supplementary Information

SI1. Phase space reconstruction

Phase space was reconstructed for the discretely sampled time series of the state variables from the ME-R model (i.e., succinate, membrane potential, and mitochondrial H_2O_2). For phase space reconstruction, y(t) = [x(t), x(t + τ), x(t + 2τ)…].The time lag (τ) value was determined from the first minimum of the non-linear correlation function called average mutual information, and was computed using MutualInfo 0.9 package[1, 2] in MATLAB R2017a. Mutual information measures the extent of which a variable, *s(n)*, is related to itself at a given time lag (*s(n + τ)*) according to the following expression [3]

$$I(\tau) = \sum_{s(n),s(n+\tau)} P\big(s(n), s(n+\tau)\big) \log_2 \left[\frac{P(s(n), s(n+\tau))}{P\big(s(n)\big)P(s(n+\tau))}\right]$$

For the time series studied herein (Fig. 1C, panel f, and Fig. 3A-D) the time lag used for the phase space reconstruction was the first minimum detected by the average mutual information are represented with red arrows in Supplementary Figs. S2B and S3E-H, respectively.

The appropriate embedding dimension is calculated according to the false-nearest neighbor technique to determine the complete unfolding of the geometrical structure (i.e., points laying close to one another in the space of the x(t) vectors because of their dynamics but not of their projection)[4]. Following[5] and using MATLAB R2017a, we examined the nearest neighbor in phase space of the vector y(k) with time label k. This method searches for points in the data set which are neighbors in embedding space of *m* dimensions, but which should not be neighbors since their future temporal evolution is too different. For each point of the time series ($s_n^{(m)}$) the closest neighbor in *m* dimensions is determined; then the distance between these two points in m+1 dimensions and in *m* dimensions is estimated. If the ratio between these distances is larger

than a threshold (σ/r), the neighbor was false, given the threshold is large enough to allow for exponential divergence due to deterministic chaos.

$$X_{fnn}(r) = \frac{\sum_{n=1}^{N-m-1} \Theta\left(\frac{\left|s_n^{(m+1)} - s_{k(n)}^{(m+1)}\right|}{\left|s_n^{(m)} - s_{k(n)}^{(m)}\right|}\right) \Theta\left(\frac{\sigma}{r} - \left|s_n^{(m)} - s_{k(n)}^{(m)}\right|\right)}{\sum_{n=1}^{N-m-1} \Theta\left(\frac{\sigma}{r} - \left|s_n^{(m)} - s_{k(n)}^{(m)}\right|\right)}$$

where $s_{k(n)}^{(m)}$ is the closest neighbor to s_n in *m* dimensions is determined, i.e. *k*(*n*) is the index of the time series element *k* different from *n* for which $|s_n - s_{k(n)}|$= min. Θ represents a step function, where the first step function in the numerator is unity, i.e. if the closest neighbor is false.

To determine the complete geometric unfolding of the attractor its embedding dimension was calculated according to the false-nearest neighbor (FNN) technique[4]. Green arrows in Supplementary Figs. S2C and S3I-L show the embedding dimension at which the percentage of global FNN dropped to zero.

References

1. Peng H. MutualInfo 0.9 package. (ed^(eds). MutualInfo 0.9 package (2002).

2. Peng H, Long F, Ding C. Feature selection based on mutual information: criteria of max-dependency, max-relevance, and min-redundancy. *IEEE Transactions on Pattern Analysis and Machine Intelligence* **27**, 1226-1238 (2005).

3. Abarbanel HDI. *Analysis of observed chaotic data*. Spinger-Verlag New York, Inc. (1996).

4. Kurz FT*, et al.* Network dynamics: quantitative analysis of complex behavior in metabolism, organelles, and cells, from experiments to models and back. *Wiley Interdiscip Rev Syst Biol Med* **9**, (2017).

5. Clauset A, Grigg N, Lim MT, Miller E. Chaos you can play in. *Proceedings of the Santa Fe Institute Complex Systems Summer School, NM*, (2003).

Table S1. State variables initial condition used in model simulations at Shunt 0.04, SOD1 9.7 10^{-5}mM and SOD2 0.0216733mM in the presence of external superoxide perturbation with a 10^{-7}mM amplitude and 3 10^{4}ms period.

State variable	Value	Unit
**Mitochondrial matrix Ca^{2+}**	2.98104994292313 10^{-5}	mM
**Mitochondrial matrix ADP**	0.0156859125787313	mM
**Mitochondrial membrane potential**	162.202747298642	mV
**Mitochondrial matrix NADH**	0.411785650760981	mM
**Mitochondrial matrix H^{+}**	2.57631270090633 10^{-5}	mM
**Mitochondrial matrix Pi**	4.50164417980960	mM
**Isocitrate**	0.0270952847289763	mM
**α-ketoglutarate**	0.131876285011142	mM
**Succinyl CoA**	0.0730662997871766	mM
**Succinate**	0.0966627229374357	mM
**Fumarate**	0.0911190663279036	mM
**Malate**	0.0741941998227697	mM
**Oxaloacetate**	0.0213476720890390	mM
**Mitochondrial matrix Na**	0.0886973771793458	mM
**Mitochondrial matrix Superoxide**	4.86321877212292 10^{-7}	mM
**Extra-matrix Superoxide**	1.27660380915538 10^{-5}	mM
**Mitochondrial matrix hydrogen peroxide**	0.0648607486044837	mM
**Extra-matrix hydrogen peroxide**	7.87037712415919 10^{-5}	mM
**Mitochondrial matrix GSH**	0.0132644286633020	mM
**Extra-matrix GSH**	0.0106963237449000	mM
**Mitochondrial matrix GSSG**	2.96452362147404	mM
**Mitochondrial matrix TrxSH2**	0.00346253699370737	mM
**Extra-matrix TrxSH2**	0.00139800603376690	mM
**Mitochondrial matrix PSSG**	0.000999987195551880	mM
**Extra-matrix PSSG**	0.000959626062408513	mM

Legend for figures

Figure S1. Scheme of the two-compartment ME-R model accounting for ionic, energetic and redox processes, their interactions, and transport between compartments

The ME-R model accounts for oxidative phosphorylation (OxPhos) and matrix-redox-based processes including the tricarboxylic acid (TCA) cycle and major antioxidant systems present in matrix and extra-matrix compartments. In addition to energy metabolism and ion transport (H^+, Ca^{2+}, Na^+, Pi), the model accounts for superoxide ($O_2^{\cdot-}$) generation in the mitochondrial electron transport chain from both complex I- and complex II-derived electron transport. $O_2^{\cdot-}$ is dismutated to hydrogen peroxide (H_2O_2) by SOD2 or can be transported as such to the extra-matrix compartment through the inner membrane anion channel (IMAC), where it can be scavenged by SOD1. Marked in red are the model parameters related to SOD1, SOD2 and the fraction of electrons from respiration diverging toward $O_2^{\cdot-}$ ("Shunt") which are main modulators of mitochondrial redox-energy dynamics (35)

Grey oscillatory line represents external sinusoidal oscillatory superoxide perturbation which was imposed to evaluate the effects of perturbing mitochondrial dynamics. *Key to symbols*: $\Delta\Psi_m$ is represented by concentric circles with an arrow across.

Figure adapted from Kembro et al (2013).

Figure S2. Mitochondrial complex oscillatory dynamics is observed in the absence of superoxide perturbation

A) Power Spectrum Analysis, (B) Mutual Information, and (C) False Nearest Neighbor (FFN) analyses performed on the same time series represented in Fig. 1C, panel f (SOD2=0.02167268014mM). A time lag of 5sec, corresponding to the first minimum in the mutual information function (red arrow in (B)), and embedding dimension of 15 (black arrow), were used for attractor reconstruction in (D). Note that due to the large embedding dimension (i.e., > 3D), the attractor does not completely unfold in 3D. (E) Phase space plots performed for the same SOD2 concentrations. Key to symbols: Succ, Succinate, $\Delta\Psi_m$, membrane potential, H_2O_2i, extra-mitochondrial H_2O_2.

Figure S3. Additional characterization of chaotic dynamics

(A-D) Power Spectrum Analysis, (E-H) Mutual Information, and (I-L) False Nearest Neighbor (FFN) analyses performed on the same time series represented in Figure 3 for SOD2 concentrations (in mM) (a, e, i) 0.013; (b, f, j) 0.016; (c, g, k) 0.0164, and (d, h, l) 0.0216733.

For all time series Shunt = 0.04, SOD1 = 9.7 10^{-5} mM, and external superoxide perturbation: amplitude = 1 10^{-7} mM, period = 30sec. Insets for panels B and D represent the PSA results plotted on a double log scale.

Figure S4. Sensitivity of chaotic attractor dynamics to changes in mitochondrial antioxidant capacity

The sensitivity of chaotic behavior was explored as a function of changes in matrix SOD2 concentration for the time series shown in Fig. 3B, D. (A, C) reconstructed attractor; (B, D) phase space plots performed for SOD2 concentrations (in mM) 0.013 (A, B) and 0.0164 (C, D). As in Figures 3 and 4 model-simulated time series were calculated with Shunt = 0.04, SOD1 = 9.7 10^{-5}mM. External superoxide perturbation: amplitude = 1 10^{-7}mM, period = 30sec. Succ: succinate; $\Delta\Psi_m$, membrane potential; H_2O_2m, mitochondrial hydrogen peroxide.

Figure S5. Bifurcation diagrams of Succinate concentration as a function of Cu,Zn superoxide dismutase (SOD1) concentration or the Shunt as bifurcations parameters

Transition from a fixed point to a limit cycle through successive period doubling is clearly observed for both parameters. Maximum and minimum values of the steady state oscillations are represented in red and blue, respectively. Parameters: SOD2 = 0.0216726976mM without external superoxide perturbation (amplitude and period = 0). In (A) Shunt = 0.04, and in (B) SOD1 = 9.7 10^{-5}mM.

Figure S6. Exogenous, steady oscillatory superoxide perturbation elicits chaotic dynamics depending on the state variable

Bifurcation diagrams of H_2O_2 (A), and GSH (B) dynamics as a function of SOD2. Notice the transition from a fixed point to a limit cycle followed by successive period doublings leading to chaotic dynamics. Of note is that for SOD2 concentrations above 0.025mM GSH exhibits relatively less dynamics than Succ and H_2O_2. Maxima and minima values of steady state oscillations are represented in red and blue, respectively. The model parameters utilized in these simulations were the same as those used in Figure 2A.

Figure S7. Amplitude delay plots in non-chaotic and chaotic dynamics

Amplitude delay plots of Succ were estimated under 4 different parametric combinations. Blue (SOD 0.0216733 mM; SOD1 9.7 10^{-5} mM) and black (SOD2 0.0216726976 mM; SOD1 8.9 10^{-5}mM) open circles are from chaotic attractors while red (SOD2 0.013 mM; SOD1 9.7 10^{-5} mM) and green (SOD2 0.0164 mM; SOD1 9.7 10^{-5} mM) circles correspond to limit cycle attractors.

Figure S8. Entrainment at amplitudes 10^{-6}mM in extramitochondrial forced-superoxide oscillations

Entrainment in state variables of mitochondrial function was analyzed through pair-wise estimation of the correlation between periodograms of each variable as obtained with Power Spectrum Analysis (PSA). Displayed are the PSA of (A) succinate, (B) Oxaloacetate, and (C) extra-mitochondrial reduced thioredoxin, $TrxSH_2$. (D) The correlation matrix obtained from the power spectra of model variables is depicted. Strong (shown in red) correlations between variables indicate main shared frequencies. Compared to Figure 6, notice the predominance of red tones and the absence of dark blue tones (low correlations) due to the relatively simple oscillation generated by entrainment as displayed in Figure 5B, top panel; period of forcing oscillation 200 s.

Figure S9. Scheme of the dependence of redox perturbation-triggered chaotic dynamics on ROS generation and SODs compartmentation

Depicted in orange are the "edge" domains of complex oscillations (i.e. sum of multiple sinusoidal oscillations of different frequencies and amplitudes), chaos-free, in the absence of redox perturbation (left panel) and coexistence of chaos and complex oscillations in the presence of redox perturbation (right panel), delimiting physiological from pathological mitochondrial function (represented in blue or violet, respectively). The relative position of the "edge" domain in the graph depends on the interplay between the antioxidant capacity given by SOD1 in the periplasmic-cytoplasmic (extra-mitochondrial), SOD2 in the matrix compartments, and ROS generation by the respiratory chain.

Fig. S10. High amplitude superoxide perturbations lead to mitochondrial function impairment

Amplitudes (a) of 10^{-5} mM elicit oscillations around values of A) $\Delta\Psi_m$ ~ 0 mV, B) NADH 99% oxidized, and C) Succinate is near depletion, indicating severe mitochondrial dysfunction for a wide range of periods from 1sec to 1000sec. Periods (P) of 30 (red lines) and 100s (black lines) are displayed as representative examples. Simulations showed that higher amplitudes of the perturbation at 10^{-4} mM (green lines) are incompatible with mitochondrial function. Model-simulated time series were calculated with SOD2=0.0216733, SOD1=9.7 10^{-5} mM, and Shunt = 0.04.

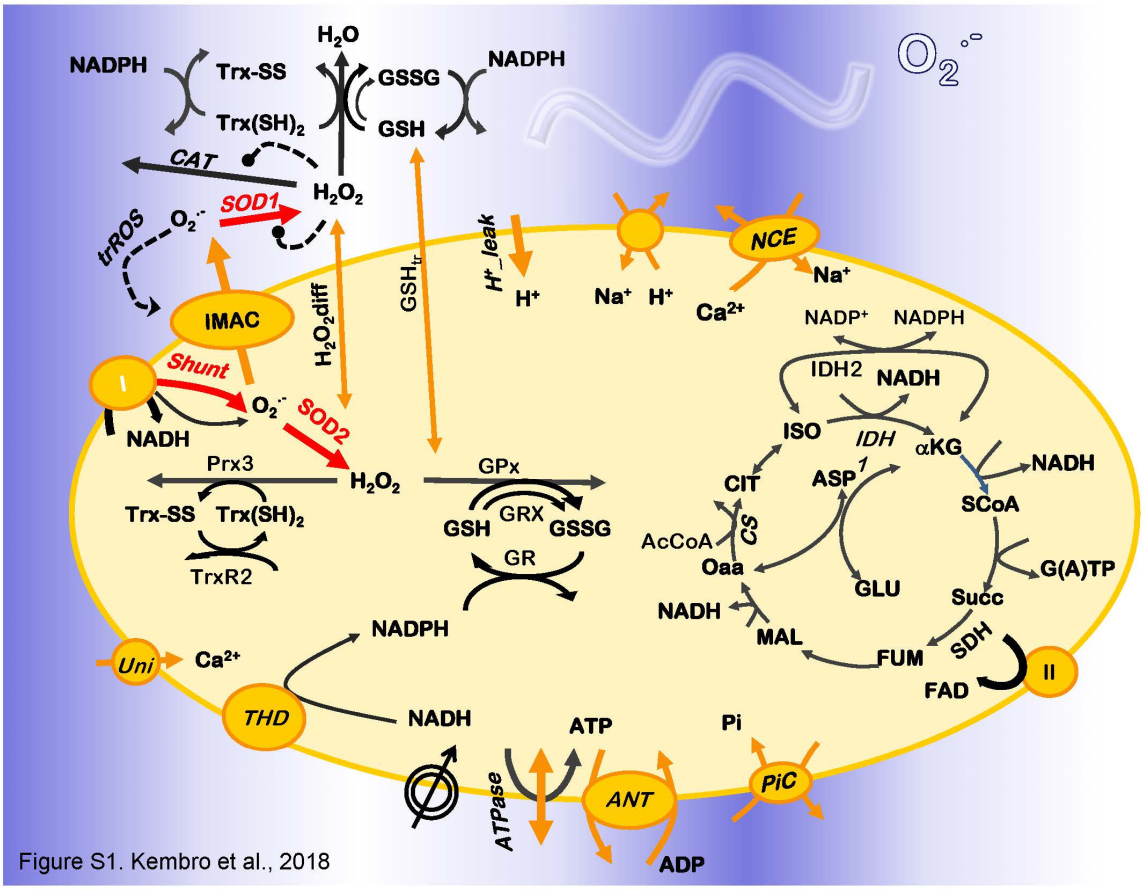

Figure S1. Kembro et al., 2018

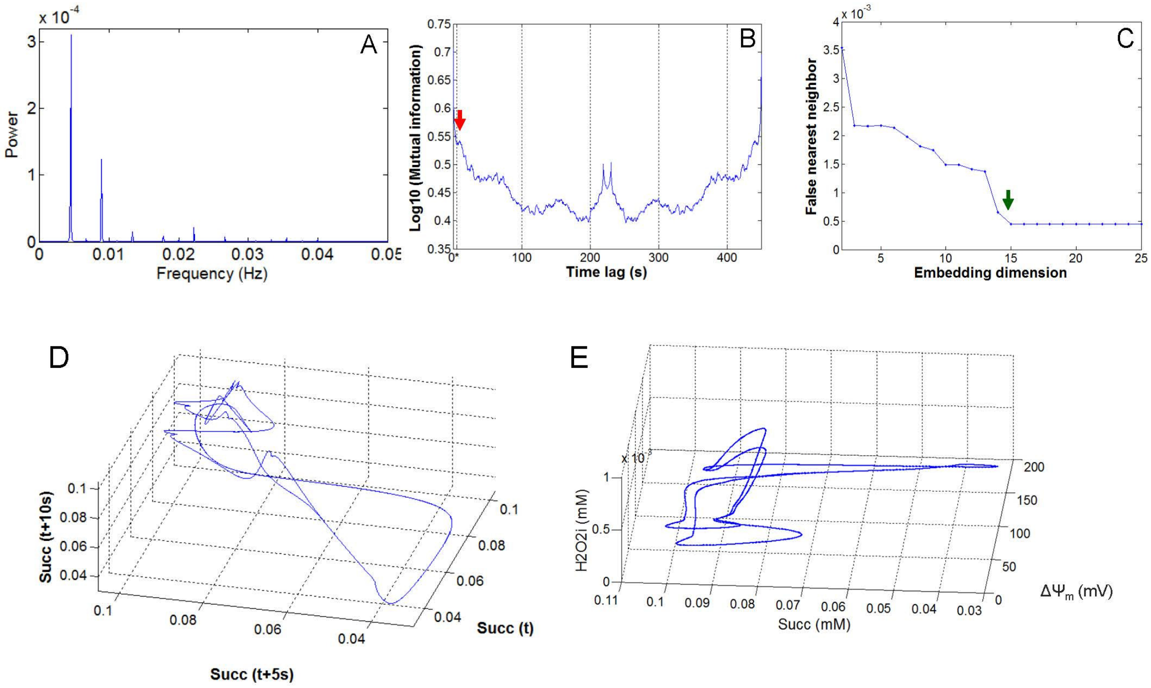

Figure S2. Kembro et al., 2018

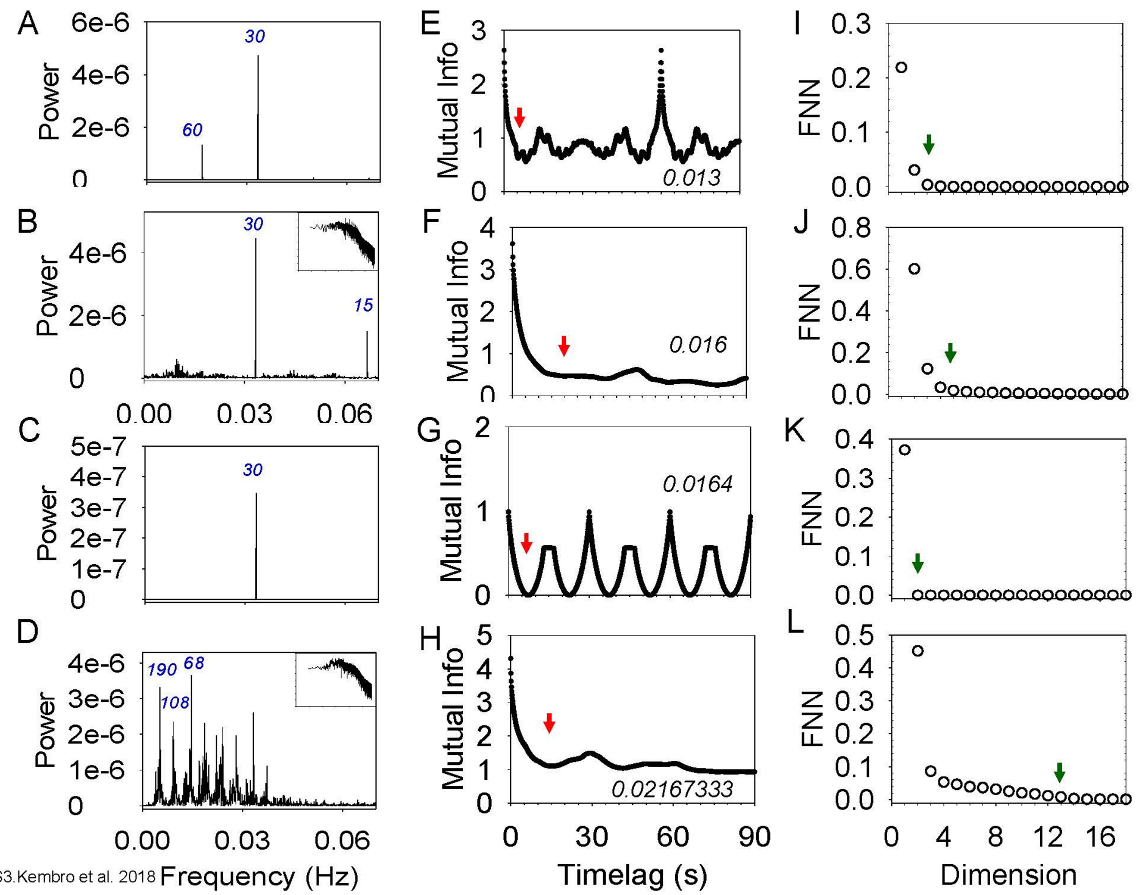

Fig. S3.Kembro et al. 2018

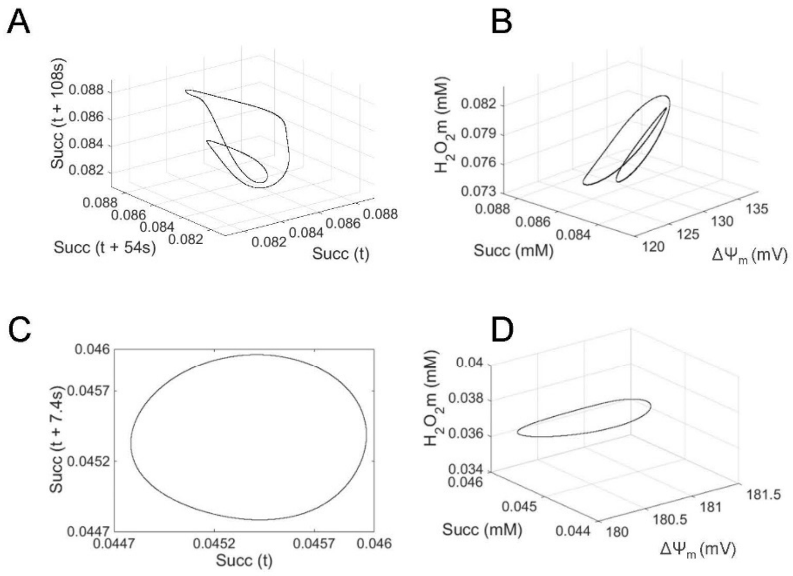

Figure S4. Kembro et al., 2018

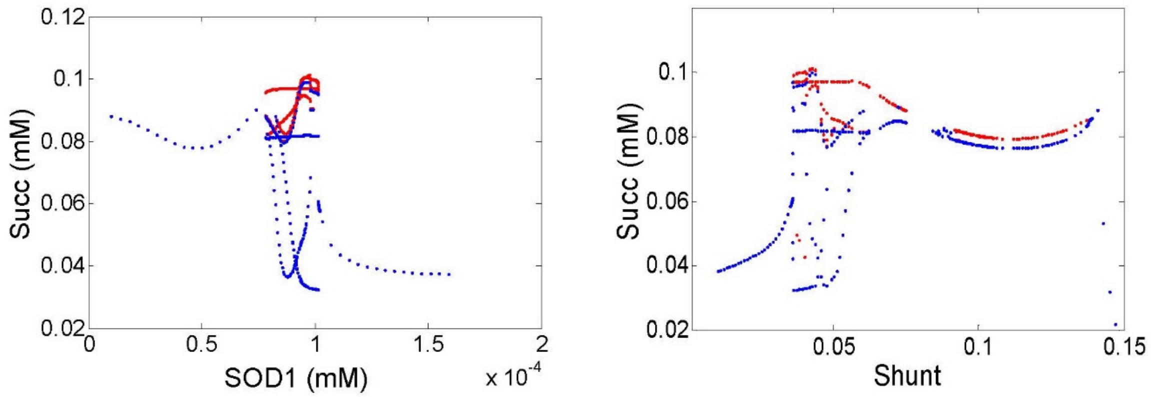

Figure S5. Kembro et al., 2018

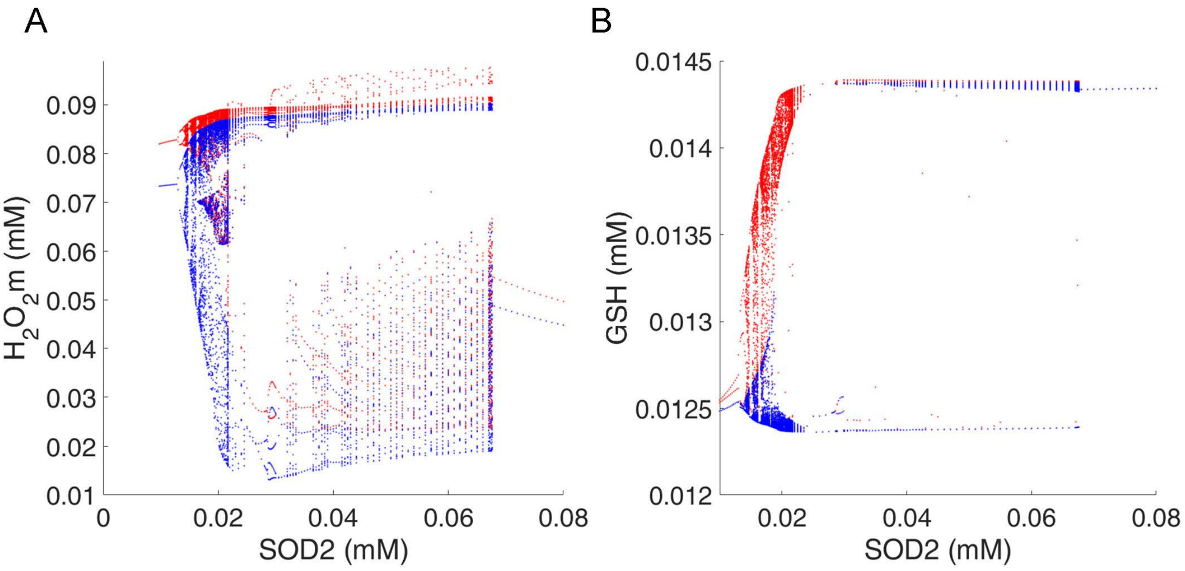

Figure S6. Kembro et al., 2018

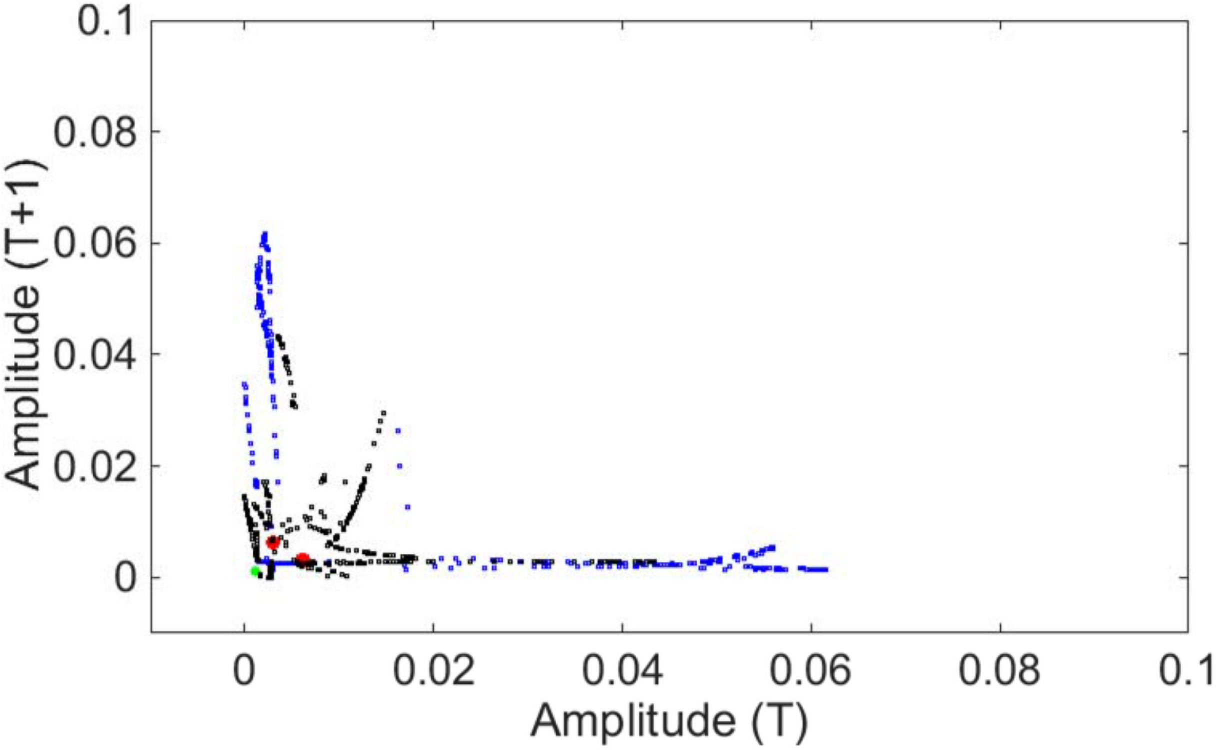

Figure S7. Kembro et al., 2018

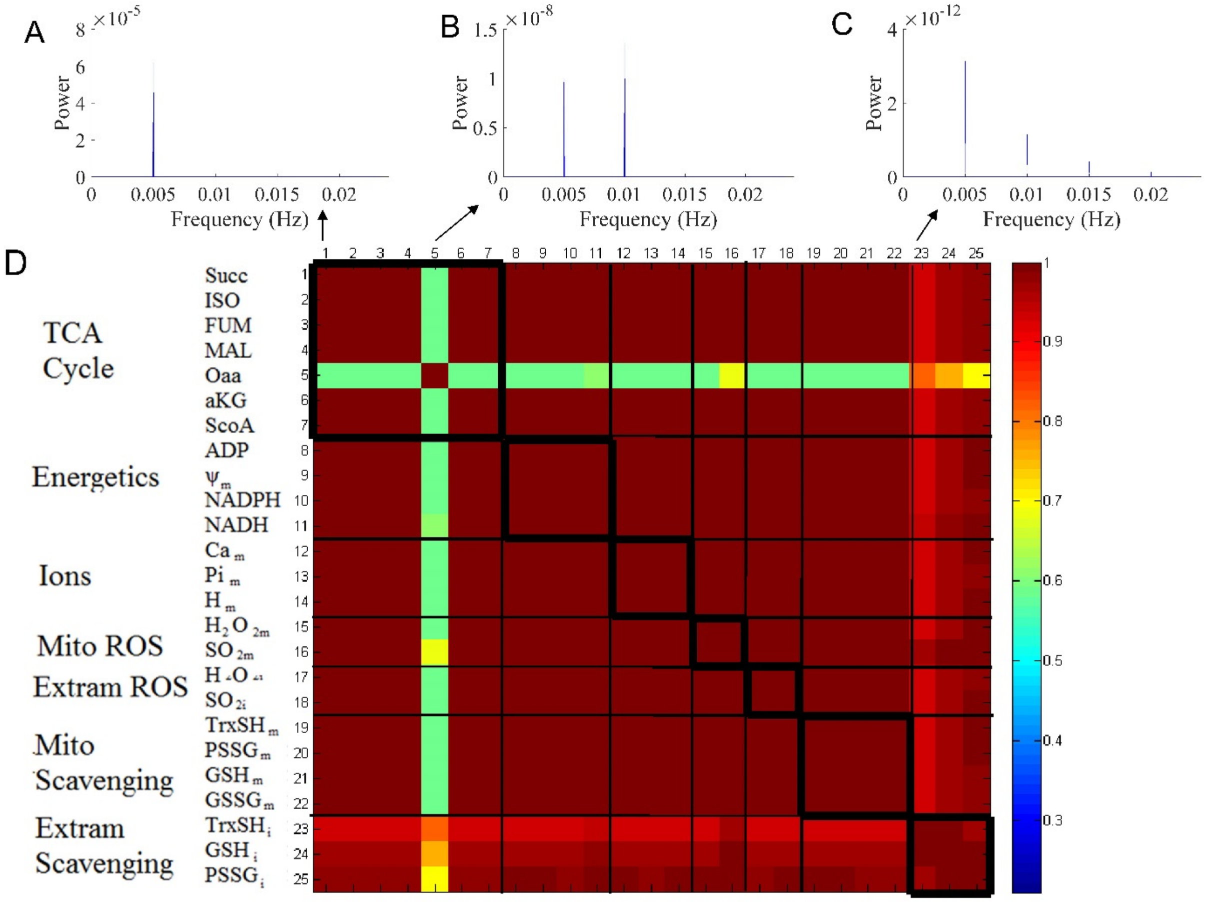

Figure S8. Kembro et al., 2018

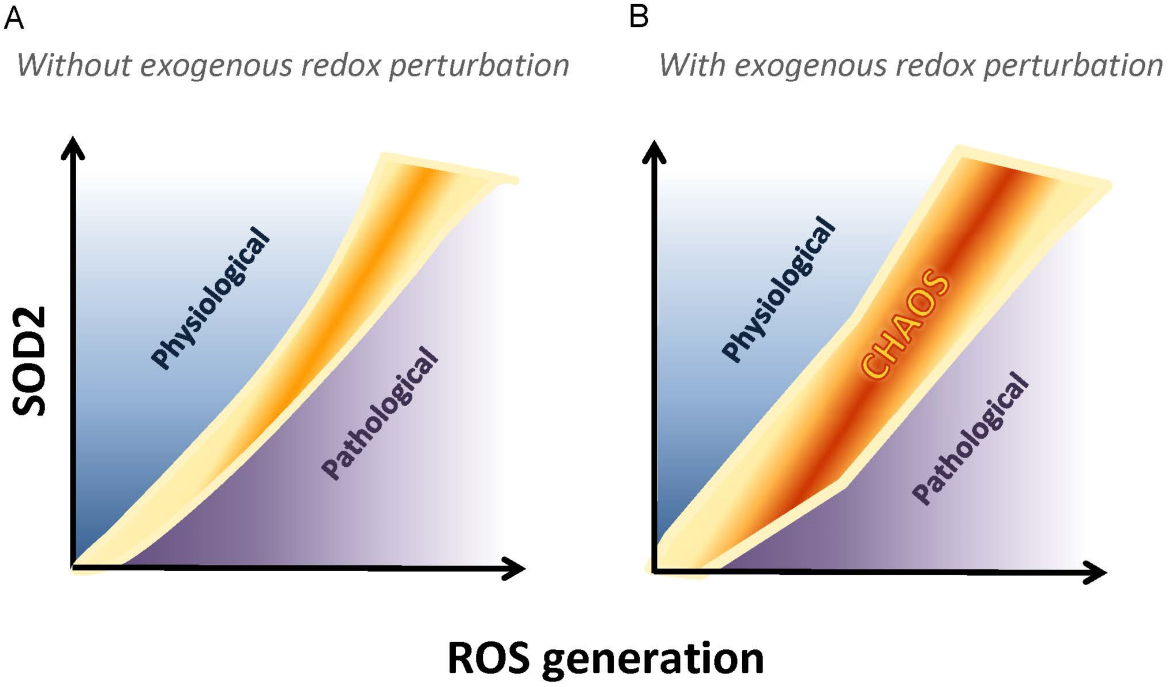

Figure S9. Kembro et al., 2018

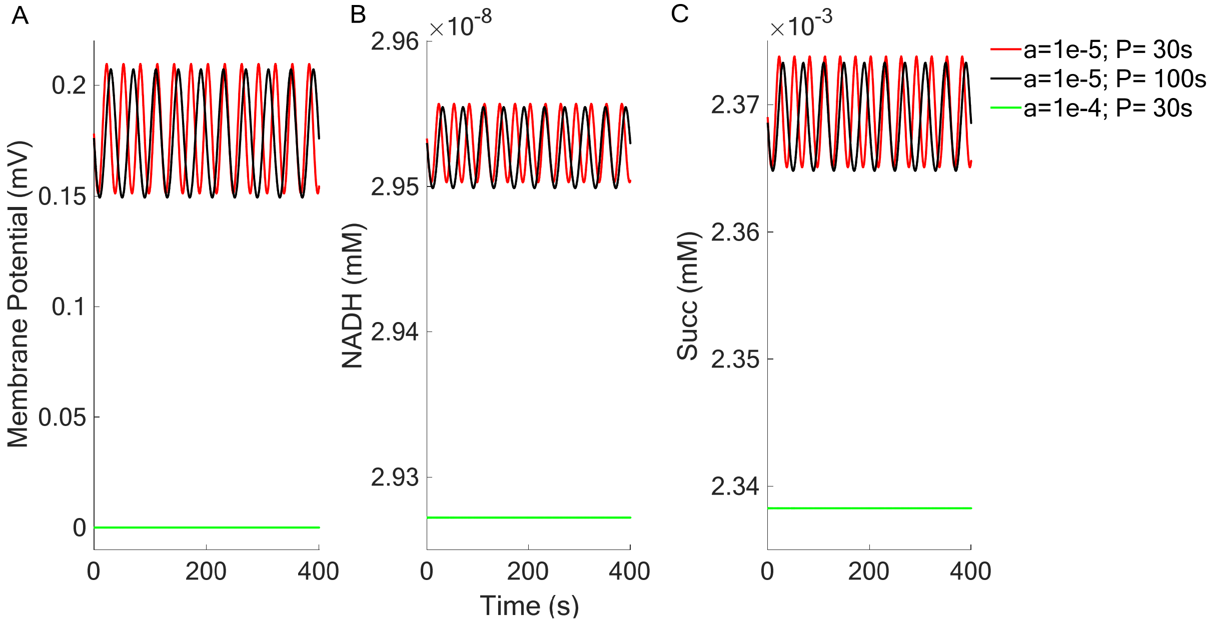

Figure S10. Kembro et al., 2018
